# Case of Croup

**Published:** 1854-05

**Authors:** J. W. Minor

**Affiliations:** Surgeon of Brooklyn City Hospital


Case of Croup.—Tracheotomy.—Successful Result.—By J. W. Minor, M. D., Surgeon of Brooklyn City Hospital.—C. A.; male, æt. 18 months, was attacked with croup, Tuesday, Dec. 20, 1853. Was promptly and efficiently treated by an intelligent parent, with the remedies usually resorted to, and generally successful in domestic practice. The next day (Wednesday) he appeared to be as well as usual, except a little hoarseness and occasional cough, which was scarcely heeded; though from the subsequent development of the disease, they must have been of such a character as would have excited alarm in an educated ear. As night approached, the hoarseness and cough increased rapidly, with all the well marked characteristics of this formidable disease. The remedies which had previously produced relief were again resorted to; but although they exercised their full specific actions, there was no abatement of the symptoms.

On Thursday evening, Dr. James Crane, of this city, was sent for, and found him laboring under all the well-marked symptoms of the disease, viz: dyspnœa, dry husky voice, and cough, &c. Ordered calomel and antimony every two hours. The antimony had the desired effect, producing free vomiting. About 3 o'clock, A. M., Dr. Crane was again summoned, but being engaged with anothed case, Dr. C. R. McClellan was called. He found all the symptoms much aggravated; ordered tincture of sanguinaria in addition to calomel and antimony, but without any visible effect. Was again seen by Dr. Crane about 9 o'clock, A. M., on Friday; at which time the symptoms were so much aggravated as to induce him to request a consultation with Dr. McClellan. The urgency of the case led to the opinion that further medical treatment would be of no avail, and that surgical interference would probably be required.

With this view I was summoned between 9 and 10 o'clock, A. M. On my arrival, the little sufferer was laboring for breath, with the mouth and nostrils dilated, and muscular apparatus of respiration in the most intense state of action; the face and neck suffused with carbonated blood; the breathing stridulous, and voice husky; in short, presenting all the most pressing indications for speedy surgical interference, or else a rapid termination in death.

This, together with a brief statement of the previous history of the case by Dr. Crane, induced me to fully concur with them in the opinion that tracheotomy alone could afford a chance for life; and I proceeded to operate with the least possible delay.

The operation was performed in the usual manner, and presented no features worthy of remark, except in the difficulty in cutting through the rings of the trachea, the delay produced by which, with the violent struggles of the child, had nearly caused asphyxia.

At one time we thought that the little patient must die, the pulse being scarcely perceptible, and the respirations faint and few; but by keeping him in an upright position, with the shoulbers alternately elevated and depressed, and by the administration of brandy and ammonia, the respiration was gradually restored.

Previous to the introduction of the tube, the posterior surface of the trachea was observed to be of a yellowish white or buff color, and led me to believe that the inflammation had reached that point, though the subsequent course of the case would favor the opinion that it had been confined to the larynx.

The clearing up of the purple hue of the face and neck, as the fullness and frequency of the inspirations furnished the blood with oxygen, was most striking and satisfactory. A tube was introduced, and retained by tapes around the neck. I may here mention that the one which was first introduced gave us some trouble, from not having the rim sufficiently broad and long, in consequence of which it could not be kept steady; my own canula was subsequently introduced, which having ruch a rim, answered very well.

The calomel and Dover's powders, which had been previously ordered, were continued, and directions left to clear out the tube with a moistened feather from time to time, to prevent the hardened crust of mucus which accumulates and obstructs it under such circumstances.

Saw him again the same evening, and found no material change except some febrile reaction, but not more than might be expected. Respiration easy and comfortable, slight mucous râles in the bronchial tubes. The extreme fretfulness and irritability of the child prevented a very accurate examination. Continued powders.

Saturday, 24th. Child passed a tolerable night, pulse accelerated, cheeks flushed, and some heat of skin.

The mother informed me that about midnight there had been discharged through the wound, *above the tube*, a tough, tenacious "phlegm," after which the air seemed to pass entirely *per vias naturales*, the opening in the convexity of the tube allowing it to do so, illustrating the importance of such an arrangement, permitting the returning functions to be gradually and steadily resumed, as the obstruction is removed.

5, P. M. Still some febrile reaction, respiration perfectly easy, and a little accelerated, but not more than would be the case with the same vascular disturbance under other circumstances. Inasmuch as the larynx seemed fully competent to perform its functions, and the trachea seemed almost free from disease, I removed the tube, as it was no longer needed, and would merely act as a foreign body. Fearing, however, that if the wound were closed too soon, a subsequent return of obstruction (in larynx) might take place, it was kept moderately dilated.

Sunday 25th. The same powders were continued. Still some vascular disturbance, but not more than necessary for the purpose of reparation. Wound perfectly open, permitting a clear view of the posterior lining of the trachea; but strange to say, scarcely any air escapes from it. Why is this? It is only in coughing that air in any volume escapes. Directed to continue powders.

Monday, 26th. Doing well, brought edges of wound together with adhesive straps, discontinued powders.

From this time the case progressed rapidly to a favorable termination, the wound closing in less than ten days from the time of the operation.—*New York Med. Times.*

